# Adolescent Sleep as a Transdiagnostic Factor: Associations Between Actigraphy-Derived Night-to-Night Sleep Metrics and Adolescent Psychopathology

**DOI:** 10.1016/j.jaacop.2024.06.001

**Published:** 2024-07-04

**Authors:** Eric M. Phillips, Emily L. Goldberg, Rebecca L. Brock, Emily R. Hamburger, Jennifer Mize Nelson, W. Alex Mason, Kimberly Andrews Espy, Timothy D. Nelson

**Affiliations:** a University of Nebraska-Lincoln, Lincoln, Nebraska; b Wayne State University School of Medicine, Detroit, Michigan

**Keywords:** general factor of dysregulation and psychopathology, intraindividual sleep variability, P-factor, sleep, transdiagnostic

## Abstract

**Objective:**

This study examined associations between actigraphy-derived sleep metrics (ie, intraindividual sleep variability, average sleep duration, bedtime, and waketime) and psychopathology to discern their roles as potential transdiagnostic factors related to psychiatric problems during adolescence.

**Method:**

In a sample of 238 adolescents (aged 14-18 years; 0.4% Asian American, 4.0% Black, 22.0% Multiracial, and 73.5% White; 53.4% female) oversampled for socioeconomic risk, we used a bifactor s-1 model of psychopathology, with emotion dysregulation as the reference indicator, to model the general factor of dysregulation and psychopathology (GF-DP) as well as specific internalizing and externalizing factors. We used multilevel structural equation modeling with the Markov chain Monte Carlo procedure to model latent means and intraindividual variability in nightly sleep duration, bedtime, and waketime over 14 nights, and to test whether they had general or specific associations with psychopathology. Furthermore, we examined whether sociodemographic variables moderated the associations between psychopathology and the various sleep metrics.

**Results:**

Results indicated a significant positive association between the GF-DP and intraindividual variability in sleep duration (β = 0.18, 95% CI = 0.013, 0.335). This association was consistent across multiple demographic characteristics, highlighting its broad relevance. The study did not find significant associations with specific internalizing or externalizing problems or other sleep metrics.

**Conclusion:**

Findings emphasize intraindividual variability in sleep duration as a key transdiagnostic factor in adolescent psychopathology. Targeting sleep variability could lead to more effective interventions, potentially reducing the prevalence of a broad spectrum of psychiatric disorders. Future research using diverse samples and longitudinal designs is warranted to build on these insights.

**Clinical guidance:**

• Clinicians should assess for consistency of adolescent sleep in addition to the amount of time an adolescent is sleeping. Sleep inconsistency may be more relevant to adolescents’ mental health problems than total sleep time.

• Interventions aimed at improving the consistency of young people’s sleep, such as the Transdiagnostic Intervention for Sleep and Circadian Dysfunction (TranS-C), may be beneficial in reducing diverse forms of mental health problems (eg, depression, anxiety, attention problems, oppositionality, and conduct problems).

• Improving consistency of sleep may be particularly beneficial for adolescents with comorbid mental health problems.

## Plain language summary

In this study of 238 adolescents between the ages of 14 to 18, night-to-night sleep was measured using actigraphy watches and related to parent- and adolescent-rated mental health problems. Greater inconsistency in the amount of time an adolescent slept each night was related to greater emotion dysregulation and mental health problems, including internalizing (ie, depression, anxiety, somatic problems) and externalizing (ie, oppositionality, conduct problems, hyperactivity, and inattention) problems. The average amount of sleep time and bedtime and waketime were not significantly related to adolescents’ mental health problems.

Adolescence, a period characterized by significant neurobiological changes, coincides with an increased vulnerability to psychiatric disorders,^1^ with the peak age of onset of first psychiatric disorder at approximately 14.5 years.^2^ Recent research has highlighted that many psychiatric disorders are not isolated: they frequently co-exist or have overlapping symptoms, suggesting shared underlying mechanisms.^3^^,^^4^ By identifying what is consistently present across multiple disorders (common factors) and what is unique to each (specific factors), we can gain deeper insights into the origins of these conditions. Furthermore, elucidating modifiable predictors and correlates of those factors (eg, sleep problems, diet, stress, interpersonal conflict) can guide us in developing interventions that can target multiple disorders at once or can be tailored to address the unique aspects of each condition.

Building on this understanding of overlapping psychiatric symptoms, the pervasive comorbidity and continuous nature of psychiatric problems have led some researchers to propose the existence of an underlying general factor of psychopathology,^5^ known as the *p* factor. Similar to others,^6^ in the present paper we use “*p* factor” to refer to the theoretical construct and use “general factor” to refer to the statistical construct and model. A large body of work over the last decade has underscored the reliability, validity, and utility of the general factor.^7^, ^8^, ^9^ This research demonstrates that the general factor can be effectively modeled in children, adolescents, and adults, accounting for the majority of shared variance in psychiatric symptoms.^5^^,^^10^^,^^11^ Recent insights suggest that the *p* factor, particularly in children and adolescents, is best characterized by emotion dysregulation.^11^, ^12^, ^13^ This dysregulation is postulated to emerge when reactive, bottom-up processes override an individual’s top-down regulatory resources, crucial for emotion regulation (ie, affect regulation imbalance). Such affect regulatory imbalances are believed to be the foundation of almost all forms of psychopathology.^12^^,^^14^^,^^15^ Thus, the *p* factor has been identified as a central transdiagnostic factor, holding promise to steer early interventions that could address and potentially avert long-standing psychopathological risks.^16^ However, to harness its full potential as a parsimonious transdiagnostic target, it is imperative to understand the various modifiable risk factors and shared mechanisms of the *p* factor. Furthering our understanding of the various mechanisms and risk factors that relate to the *p* factor has the potential to inform our understanding of common etiological pathways and potential malleable targets to reduce the development and maintenance of psychopathology during key developmental periods such as adolescence.

One factor that is critical to adolescent mental health, but that has thus far received limited attention in the context of the *p* factor, is sleep. Various aspects of sleep play indispensable roles in adolescent cognitive function, emotional regulation, and overall mental well-being.^17^, ^18^, ^19^ A large body of work points to the ubiquitous nature of sleep problems in psychiatric disorders.^18^^,^^20^ Studies have consistently highlighted that inadequate sleep is intertwined with nearly all prevalent forms of psychopathology during adolescence, encompassing both internalizing and externalizing disorders.^19^^,^^21^, ^22^, ^23^ This work has led various researchers to propose sleep disturbances as an underlying transdiagnostic factor related to the etiology and maintenance of psychopathology through its reciprocal links with emotion (dys)regulation and relevant neurobiological mechanisms.^18^^,^^24^^,^^25^ Relatedly, previous work using the present sample demonstrated associations between sleep/wake problems, emotion regulation strategies, and internalizing psychopathology.^26^

Unfortunately, previous research has primarily adopted a narrow focus, examining sleep disturbances in relation to specific psychiatric disorders or dimensions, largely in isolation.^27^ This is problematic for various reasons, including a lack of consideration for the inherent comorbidity and the continuous, dimensional nature of psychopathology; a reduction in statistical power; and lower reliability and validity of psychiatric diagnoses and lower-order symptom dimensions.^3^ In addition, this selective approach leaves unresolved questions: are sleep disturbances in adolescence unique to specific forms of psychopathology, or do they transcend diagnostic and dimensional boundaries? Establishing sleep disturbances as truly transdiagnostic could illuminate shared pathways and common mechanisms of psychopathology, paving the way for more parsimonious, efficient, and potentially transformative interventions that could address a broad spectrum of psychiatric disorders with a single treatment.^18^

Furthermore, most work on sleep and psychopathology has focused on mean sleep duration. However, recent work has identified intraindividual sleep variability—that is, night-to-night fluctuations in an individual's sleep patterns—to be particularly relevant to psychopathology, beyond mean sleep duration.^28^, ^29^, ^30^ For example, one study using accelerometer-derived sleep measures across 7 days in a large sample of 89,205 adults found that intraindividual sleep variability (measured as the median absolute deviation of sleep duration), but not mean sleep duration, was significantly and consistently associated with all included psychiatric diagnoses (schizophrenia spectrum disorders, bipolar disorder/mania, major depressive disorder, and anxiety disorders).^27^ This suggests that focusing solely on mean sleep duration may not provide a comprehensive understanding of the link between sleep and psychopathology. Although these findings offer compelling insights for adults, it remains an open question whether there is a similar pattern of results during adolescence. To our knowledge, no study has comprehensively examined intraindividual sleep variability as a transdiagnostic factor related to multiple forms of psychopathology in adolescence.

Understanding how intraindividual sleep variability may relate to adolescent psychopathology is further complicated by methodological challenges. The vast majority of studies that have looked at intraindividual sleep variability in adolescence have relied on simplistic methods that have been shown to have poor reliability and to result in lower power,^31^ such as computing standard deviations from the various nights of sleep data.^28^ Thus, there is a pressing need to use more refined and sophisticated analytical approaches that can more accurately capture and contextualize the dynamic nuances of sleep variability (eg, multilevel structural equation modeling [MSEM]), especially when examining its role in adolescent psychopathology. Intraindividual sleep variability might play a pivotal role, alongside mean sleep duration, in the broader landscape of mental health during the crucial developmental period of adolescence.^28^^,^^30^ Improving our understanding of this often-overlooked dimension of sleep using more sophisticated and precise methods not only challenges our conventional understandings of sleep-related disturbances but could also unlock novel sleep-focused therapeutic strategies that would be more tailored to the complexities of adolescent psychiatric disorders.

To address these gaps in the literature, the present study embarked on an innovative approach to comprehensively investigate the association between actigraphy-derived sleep metrics and the *p* factor in a sample of 238 adolescents. We hypothesized that shorter average sleep duration and higher intraindividual variability in sleep duration would be transdiagnostic factors related to the general factor of dysregulation and psychopathology, above and beyond specific internalizing and externalizing problems. In addition, we tested whether greater intraindividual variability in bedtime or waketime have positive associations with general or specific psychopathology in adolescence. Finally, we conducted exploratory analyses to determine whether sex assigned at birth, age, maternal education, or income-to-needs ratio would moderate the associations between psychopathology and the various sleep metrics. By discerning whether key developmental aspects of sleep in adolescence are indeed transdiagnostic, our findings could offer valuable insights for streamlined and impactful interventions, allowing for a unified approach to managing a diverse range of psychiatric challenges during this critical developmental period.

## Method

### Participants

A total of 238 adolescents (mean age = 15.27 years, 53.4% female) participated in the study as part of a longitudinal investigation of cognitive development beginning in preschool. In all, 337 preschoolers enrolled in the initial study in preschool; 70.62% of the original preschool sample was retained in adolescence. Missing data status during follow-up was not significantly associated with any demographic variables, suggesting no systematic pattern of missingness. The racial (73.5% White, 4.0% Black, 0.4% Asian American, 22.0% multiracial) and ethnic (13.0% Hispanic) composition of the sample matched the geographic region. Advertising locations were strategically selected to recruit families from a broad range of socioeconomic circumstances, including substantial representation from families with low income. Thus, participants of the overarching study were oversampled for sociodemographic risk at entry into the study, with 39.5% who were receiving public assistance and/or who were below the poverty line according to state and federal poverty guidelines during preschool. For comparison, 21.3% of the population in the United States received public assistance from one or more public assistance programs (eg, Medicaid, Supplemental Nutrition Assistance Program) during the years that the present sample was recruited, between 2009 and 2012.^32^

### Procedures

Adolescents participated in 2 sessions, the first of which was scheduled around their birthday when possible. Participants’ parents or legal guardians provided written informed consent for their children to participate in the adolescent phase of the study, and adolescents provided written assent. During the initial session, participants completed measures related to mental and physical health as well as a task-based battery of cognitive functioning. Participants returned approximately 2 weeks later to return actigraph devices that tracked sleep. Adolescents were compensated up to $92 for full participation with the actigraphy protocol, and adolescents and parents received gift cards for attending sessions. The University of Nebraska–Lincoln Institutional Review Board approved all study procedures.

### Measures

#### Adolescent Psychopathology

Adolescent psychopathology was measured using caregiver and adolescent reports of the withdrawn/depressed (WD; caregiver α = 0.79, adolescent α = 0.77), anxious/depressed (AD; caregiver α = 0.83, adolescent α = 0.88), somatic complaints (SOM; caregiver α = 0.66, adolescent α = 0.80) subscales of the Child Behavior Checklist for Ages 6 to 18 (CBCL 6-18) and the Youth Self Report (YSR)^33^; the attention-deficit/hyperactivity disorder (ADHD) hyperactive (caregiver α = 0.92, adolescent α = 0.84), ADHD inattentive (caregiver α = 0.90, adolescent α = 0.90), oppositional defiant disorder (ODD) (caregiver α = 0.88, adolescent α = 0.83), and conduct disorder (CD) (caregiver α = 0.69, adolescent α = 0.90) subscales of the Conners 3^34^; and the Emotion Regulation Index (ERI) (caregiver α = 0.92, adolescent α = 0.88), a well-validated measure of emotion dysregulation,^35^ from the Behavior Rating Inventory of Executive Function 2 (BRIEF 2).^36^ To construct a more accurate and unbiased estimate of psychopathology, both caregiver and adolescent reports for each psychopathology facet were standardized and aggregated prior to their inclusion as indicators in the structural models. This approach aimed to mitigate potential rater biases and to capitalize on the unique yet complementary insights provided by caregivers and adolescents about the adolescents’ mental health. By integrating these diverse perspectives, we sought to capture a consensus view of psychopathology that would reflect its multifaceted nature, thereby enhancing the robustness and validity of our psychopathology constructs within the context of our study.

#### Sleep Measures

In the present study, sleep was assessed following the procedures established by Tomaso *et al.*^37^ Participants were instructed to wear a wGT3X-BT ActiGraph device (ActiGraph) continuously on their nondominant wrist to monitor sleep for a consecutive 2-week period. Participants received a daily sleep survey at 7 pm via SMS or e-mail, in which they reported when they tried to fall asleep the previous evening and when they woke up that morning. Surveys were dispatched strategically in the evening to avoid morning conflicts, such as school, work, or sleeping late. Data processing was completed in ActiLife, Version 6.13, using the Sadeh algorithm,^38^ which has been validated for use with adolescents.^39^ Reports from daily diaries were used to resolve instances in which algorithm-derived sleep periods clearly did not align with the observed wrist movement patterns, or vice versa, when wrist movement patterns were strongly suggestive of sleep but were not detected. Nightly sleep duration in minutes, bedtime (centered at minutes before or after 12 am) and waketime (centered at minutes before or after 12 am) over the 2-week period were the focus of the current study.

Participants’ sleep data were included if at least 3 nights of sleep data were available (n = 204). Our decision to set a minimum requirement of 3 nights of actigraphy data was informed by a balance between practical feasibility and the analytical robustness of our methods. Our use of Bayesian full-information likelihood approach for handling missing data allowed us to reduce potential biases by using all observed data to inform estimates. This critical methodological choice ensured the accuracy of our intraindividual variability estimates and preserved the statistical power of our analyses by retaining a larger sample size. It is important to note that our study’s methodology and the handling of missing data contrast with traditional approaches, which may require complete data sets and could potentially exclude valuable information from participants with incomplete data. Our inclusive approach, which integrates all observed data, minimizes the potential for bias and enhances the robustness of our findings. Demographic and psychopathology differences between participants with valid sleep data and those without are presented in Table S1. The occurrence of missing/invalid sleep data was negatively associated with age (*r* = −0.148, *p* = .022), such that participants with valid sleep data tended to be older than participants without valid sleep data. There were no other statistically significant differences in demographic or psychopathology variables between participants with and without valid sleep data. Compliance for those with valid sleep data was good, with participants wearing the device for an average of 12.13 nights (SD = 2.95) of the requested 14 nights. In addition, 195 of the 204 participants (95.59%) with valid sleep data had a minimum of 3 consecutive nights, and 202 of the 204 participants (99.01%) had at least 1 weekend night.

### Data Analytic Approach

To reduce model complexity and optimize the ratio of parameter estimates to sample size, factor scores of the psychopathology factors were extracted and used in all models. Measurement models were tested using full information maximum likelihood (FIML) estimation to address missing data^40^ and the robust maximum likelihood estimator (MLR) was used to address non-normality and to obtain robust SEs. The comparative fit index (CFI), root mean squared error of approximation (RMSEA), and standardized root mean squared residual (SRMR) were computed to assess global model fit. A CFI above 0.90 was interpreted as indicating acceptable model fit, and above 0.95 interpreted as indicating good model fit. An RMSEA under 0.08 was interpreted as indicating acceptable model fit, and under 0.06 interpreted as indicating good model fit.^41^ An SRMR under 0.08 was interpreted as indicating acceptable model fit.^42^

The adolescent general factor of dysregulation and psychopathology (GF-DP) was modeled using the bifactor s-1 model^43^ with emotion dysregulation as a reference indicator. The bifactor s-1 model was selected over the symmetrical bifactor model (a bifactor model in which all indicators load on both a general factor and respective domain specific factors) because of symmetrical bifactor model’s tendencies to be limited by estimation problems, inadmissible solutions, domain-specific factors collapsing, and difficulties interpreting the meaning of the general and specific factors. The bifactor s-1 model differs from the symmetrical bifactor model in that select indicators are modeled to load exclusively on the general factor. This model facilitates the empirical testing of well-founded theoretical assumptions regarding the nature of the *p* factor. It achieves this by first defining the general factor based on a solid theoretical foundation and then using direct measurements as indicators for the general, but not the specific, factors. Such an approach addresses common challenges associated with symmetrical bifactor models, including estimation difficulties, inaccurate parameter estimates, and issues of interpretability, offering a clearer understanding as detailed by Heinrich *et al.*^43^

Emotion dysregulation was selected as the reference indicator based on an extensive body of research indicating that emotion dysregulation is a core component underlying diverse forms of psychopathology, especially during adolescence—a period marked by significant emotional and neurodevelopmental changes.^1^ This choice is supported by previous research using the present sample, which found that emotion dysregulation accounts for a significant portion of the covariance between internalizing and externalizing problems; correlates nearly perfectly with the general psychopathology factor when excluded as an indicator; and has a nomological network almost identical to that of the traditionally modeled general psychopathology factor (Phillips, unpublished data, 2024). This evidence underscores the central role of emotion dysregulation in adolescent psychopathology, justifying its selection as the reference indicator to provide a nuanced understanding of the underlying structure of psychopathology.

All psychopathology indicators were modeled to load on the general factor, with the withdrawn/depressed (WD), anxious/depressed (AD), and somatic compaints (SOM) indicators modeled to also load on the internalizing specific factor, and the ADHD inattentive (AT), ADHD hyperactive (HYP), ODD, and CD indicators modeled to load on the externalizing specific factor. Given the large association generally found between inattention and hyperactivity, the residuals of the 2 indicators were allowed to covary. In addition, consistent with previous work using the present sample (Phillips, unpublished data, 2024), modification indices suggested 2 theoretically justifiable modifications. The residual of the AD indicator was negatively associated with the adolescent externalizing problems factor, and the residual of the WD indicator was negatively associated with the residual of the HYP indictor. The negative association between anxiety and externalizing problems was theoretically consistent with previous research indicating that anxiety is negatively related to and potentially a protective factor against impulsive/externalizing problems.^44^ The negative association between a tendency to be withdrawn or depressed and the tendency to be hyperactive was also theoretically justified and consistent with previous work using the withdrawn/depressed and Conners-3 hyperactivity subscales.^45^ Thus, AD’s residual was allowed to correlate with the externalizing factor, and WD’s and HYP’s residuals were allowed to correlate with one another.

#### Multilevel Structural Equation Modeling

We implemented multilevel structural equation modeling (MSEM) using a Markov chain Monte Carlo (MCMC) procedure (ie, the “Bayesian estimator” in Mplus) to analyze daily repeated measures nested within-persons. Fundamental to MSEM is the disaggregation of variance into within-person (time-varying) and between-person (time-invariant) counterparts. MSEM using the MCMC procedure is advantageous over alternative approaches for modeling intraindividual variability (eg, traditional multilevel modeling) because it allows for the following: (1) examination of random within-person variances (eg, inconsistency in sleep) as predictors, as well as outcomes of other between-person level variables; (2) multiple outcomes in 1 step; and (3) modeling latent variables to address measurement error. Furthermore, the MSEM approach results in more precise and reliable estimates of within-person variability, particularly compared to simply computing a standard deviation or a mean square of successive differences (MSSD), as one can estimate the within-person level residual variance. For example, with repeated measures data, it is not uncommon for someone to skip an assessment or otherwise for there to be unequal intervals between measurements. By including time (eg, night) as a predictor at the within-person level, one can account for these unequal intervals, control for practice effects, and account for systematic patterns of change. Thus, the within-person level variability that is estimated more accurately reflects the extent to which there is variability around a linear trajectory of change. Collectively, these various advantages make MSEM with the MCMC procedure ideally suited for testing associations between person-level characteristics (eg, psychopathology) and intraindividual variability in a construct (eg, sleep variability over multiple nights).^46^

Analyses were conducted in Mplus 8.5 using 2 MCMC chains and the Gibbs algorithm. The occurrence of missing/invalid sleep data was associated with age, which was an exogenous predictor in all models, thus addressing this systematic missingness. The estimation method that we used is a full information approach; thus, all cases were retained for analysis despite missing data, similar to full information maximum likelihood. Thus, all 34 individuals with missing or excluded sleep data were retained in all analyses, with their sleep data considered missing. The default potential scale reduction convergence criterion was used, which concludes convergence when the potential scale reduction nears 1.0. We then doubled the number of iterations to ensure that the potential scale reduction value remained near 1.0. After the model converged, we examined the 95% credibility intervals (CI), based on posterior distributions, for each of the hypothesized effects. CIs that did not contain zero were considered “significant” (ie, 95% probability that the population parameter is not zero).

We modeled intraindividual variability in night-to-night sleep duration, bedtime, and waketime using the log-transformation approach, incorporating the log-transformed random residual variances at the between-person level (see Feng and Hancock^46^ for a detailed description and Mplus code). To account for potential unequal intervals between repeated measures, the cumulative number of nights since the actigraph was first worn was included as a predictor of the within-person sleep variables. In addition, to account for systematic differences in sleep metrics due to shifts in sleep routines from weekdays to weekends, weekend (0 = weekday, 1 = weekend) was included as a predictor of all 3 within-person sleep variables. Thus, the intraindividual sleep residual variability factors captured variability in night-to-night sleep that was not attributable to any systematic linear change or to whether it was a weekend or weeknight.

At the between-person level, the intraindividual sleep variability factors, along with average nightly sleep duration, average nightly bedtime, and average nightly waketime (6 total “sleep metrics”) were all regressed on factor scores of the GF-DP, specific internalizing factor, and specific externalizing factor. We covaried the residuals of sleep outcomes at the between- person level to account for shared measurement variance. Sex assigned at birth, age, maternal education, and income-to-needs ratio were included as control variables at the between-person level.

To examine whether various demographic characteristics moderated the associations between the *p* factor and the various sleep metrics, exploratory analyses were conducted, building off the models described above, by adding interaction effects between the GF-DP and sex assigned at birth, age, maternal education, and income-to-needs ratio, for a total of 4 interaction effects. The GF-DP and demographic variables were standardized into *z* scores before creating interaction terms. One model was run with all 4 interaction effects tested simultaneously.

To determine whether associations between the *p* factor and sleep metrics are truly transdiagnostic or primarily driven by specific forms of psychopathology subsumed by the general factor, supplementary analyses were conducted with correlated psychopathology factors (ie, internalizing, externalizing, and emotion dysregulation) in place of the general and specific factors from the bifactor model. In these models, the correlated factors were modeled as endogenous, and separate models were run for sleep duration and for bedtime and waketime.

## Results

### Descriptive Statistics

Descriptive statistics and distributions of sociodemographic and study variables are summarized in Table 1.

**Table 1:** Demographic Characteristics and Key Study Variables

Characteristics	Mean	SD		
Age	15.26	1.14		
Income-to-needs ratio	2.53	1.84		

	**n**	**%**		

**Sex (assigned at birth)**				
Female	127	53.4		
Male	111	46.6		
**Race**				
Asian	1	0.42		
Black or African American	10	4.20		
Multiracial	53	22.27		
White	174	73.11		
**Ethnicity**				
Not Hispanic or Latino	208	87.39		
Hispanic or Latino	30	12.61		
**Maternal education**				
Without HS diploma (<12 y)	7	2.94		
High school graduate without college education (12 y)	19	7.98		
Some college education (13-15 y)	105	44.12		
Degree from 4-y college or more (>19 y)	107	45.00		

Key study variables	Mean	SD	Minimum	Maximum

Sleep duration (average)	389.54	46.37	230.33	503.64
Bedtime (average)	–3.83	77.72	–169.78	266.50
Waketime (average)	463.07	70.23	289.00	703.29
Sleep duration (SD)	79.10	29.93	20.86	184.13
Bedtime (SD)	74.22	40.07	15.00	262.87
Waketime (SD)	91.92	37.53	20.23	213.96
General factor	0.01	0.95	–1.73	3.28
Internalizing	0.01	0.84	–1.74	3.66
Externalizing	–0.01	0.86	–1.74	3.30

Note: Descriptive statistics for psychopathology variables are derived from factor scores and therefore are on a standardized scale. Descriptive statistics for sleep variables are based on scores derived from the means and SDs across available nights of sleep data for each participant. The method to create these scores differs from the method used to estimate participants’ latent scores in the main analyses.

### Measurement Model

Model fit was good for the bifactor s-1 psychopathology model(χ^2^ [10] = 7.56, *p* = .67, CFI = 1.00, RMSEA = 0.000, SRMR = 0.018). Standardized factor loadings are presented in Table 2. Emotion dysregulation was the strongest indicator of the general factor, with a standardized factor loading of 0.92. Factor loadings for the general factor were salient (standardized factor loadings >0.50 for all indicators) across indicators from both internalizing and externalizing psychopathology domains. The specific externalizing and internalizing problems factors were also well defined, with median factor loadings greater than 0.50 for both factors.

**Table 2:** Bifactor s-1 Model Standardized Factor Loadings

Indicator	General		Internalizing		Externalizing	
	Estimate	*p*	Estimate	*p*	Estimate	*p*
Anxious/depressed	0.641	.001	0.641	.001		
Withdrawn/depressed	0.573	.001	0.584	.001		
Somatic problems	0.569	.001	0.459	.001		
Oppositional defiance	0.719	.001			0.590	.001
Conduct problems	0.509	.001			0.611	.001
Hyperactivity	0.558	.001			0.428	.001
Inattention	0.727	.001			0.246	.001
Emotion dysregulation	0.918	.001				

### Multilevel Structural Equation Modeling

Intraclass correlations suggested that 17% of the total variance in nightly sleep duration was at the between-person level (intraclass correlation coefficient [ICC] = 0.17) with the remaining 83% at the nightly (within) level; 42% of the variance in nightly bedtime was at the between-person level (ICC = 0.42), with the remaining 58% at the nightly level; and 29% of the variance in daily waketime was at the between-person level (ICC = 0.29), with the remaining 71% at the daily level.

Results indicated that the general factor of dysregulation and psychopathology (GF-DP) was significantly positively associated with intraindividual sleep duration variability, such that individuals higher in general psychopathology tended to have more variable, or less consistent, sleep duration from night to night (β = 0.18, 95% CI = 0.013, 0.335). Intraindividual sleep duration variability was not significantly associated with specific internalizing or externalizing problems. Furthermore, unlike variability in sleep duration, intraindividual variability in bedtime and waketime were not significantly associated with any psychopathology factor. Average sleep duration, bedtime, and waketime were not significantly associated with any psychopathology factor. Unstandardized coefficients are presented in Table 3.

**Table 3:** Unstandardized Parameter Estimates for Dysregulation and Psychopathology (GF-DP) Sleep Model

Parameter	Unstandardized			
	Estimate	Posterior SD	95% CI	95% CI
			LL	UL
**Sleep duration variability**				
General factor	**0.121**	**0.056**	**0.009**	**0.229**
Internalizing specific factor	0.051	0.064	–0.074	0.178
Externalizing specific factor	–0.005	0.071	–0.142	0.134
Age	0.015	0.047	–0.074	0.109
Child sex assigned at birth	–0.020	0.124	–0.269	0.215
Maternal education	**–0.085**	**0.029**	**–0.140**	**–0.027**
Household income-to-needs ratio	–0.019	0.035	–0.089	0.049
**Bedtime variability**				
General factor	0.058	0.077	–0.089	0.211
Internalizing specific factor	0.125	0.088	–0.051	0.291
Externalizing specific factor	0.098	0.094	–0.083	0.285
Age	0.014	0.063	–0.103	0.147
Child sex assigned at birth	**0.328**	**0.158**	**0.015**	**0.633**
Maternal education	–0.055	0.036	–0.128	0.014
Household income-to-needs ratio	–0.064	0.047	–0.159	0.026
**Waketime variability**				
General factor	0.127	0.077	–0.021	0.286
Internalizing specific factor	0.123	0.089	–0.050	0.295
Externalizing specific factor	–0.037	0.097	–0.229	0.153
Age	0.071	0.066	–0.060	0.198
Child sex assigned at birth	0.157	0.16	–0.158	0.471
Maternal education	–0.032	0.038	–0.108	0.040
Household income-to-needs ratio	–0.064	0.048	–0.158	0.030
**Mean sleep duration**				
General factor	3.434	3.431	–3.148	10.201
Internalizing specific factor	–4.389	3.955	–12.030	3.386
Externalizing specific factor	–3.371	4.208	–11.485	5.003
Age	–4.770	2.979	–10.588	1.122
Child sex assigned at birth	**–27.798**	**7.221**	**–41.919**	**–13.482**
Maternal education	1.762	1.599	–1.416	4.849
Household income-to-needs ratio	1.399	2.136	–2.804	5.599
**Mean bedtime**				
General factor	6.603	5.644	–4.494	17.606
Internalizing specific factor	7.973	6.444	–4.588	20.650
Externalizing specific factor	–9.731	7.076	–23.398	4.221
Age	**14.436**	**4.870**	**4.987**	**24.017**
Child sex assigned at birth	**39.890**	**11.968**	**17.286**	**64.310**
Maternal education	–2.784	2.708	–8.165	2.455
Household income-to-needs ratio	–6.372	3.545	–13.167	0.659
**Mean waketime**				
General factor	3.633	5.383	–7.279	13.875
Internalizing specific factor	3.490	6.163	–8.351	15.757
Externalizing Specific Factor	–10.355	6.650	–23.761	2.323
Age	8.131	4.604	–1.050	16.940
Child sex assigned at birth	17.699	11.251	–3.776	40.223
Maternal education	–1.901	2.573	–6.777	3.283
Household income-to-needs ratio	–2.755	3.387	–9.472	3.840

Note: Coefficients in boldface type are significant. LL = lower limit; UL = upper limit.

Exploratory analyses indicated no significant moderation effects, suggesting that the associations between the GF-DP and the various sleep metrics did not vary as a function of sex assigned at birth, age, maternal education, or income-to-needs ratio.

Supplemental analyses with the correlated psychopathology factors indicated that the association between intraindividual sleep duration variability and psychopathology is in fact transdiagnostic, with intraindividual sleep duration variability being significantly associated with internalizing problems (β = 0.22, 95% CI = 0.037, 0.403), externalizing problems (β = 0.21, 95% CI = 0.007, 0.394), and emotion dysregulation (β = 0.22, 95% CI = 0.040, 0.412). Consistent with the GF-DP results, no other sleep metric was significantly associated with any psychopathology factor.

## Discussion

The present study sought to investigate the association between psychopathology and actigraphy-derived sleep metrics during the pivotal developmental period of adolescence. The results indicated a small but significant positive association between adolescent general psychopathology and intraindividual variability in sleep duration over 14 nights. Specifically, adolescents who scored higher in general psychopathology demonstrated less consistent sleep patterns from night to night. This association did not extend to specific internalizing or externalizing problems from the bifactor s-1 model (ie, internalizing and externalizing factors independent of common psychopathology/emotion dysregulation variance), nor did it hold for other sleep metrics such as bedtime, waketime, or average sleep duration. Importantly, these associations were consistent across age, sex assigned at birth, maternal education, and income-to-needs ratio. This consistency underscores the broad relevance of our findings, indicating that the relationship between sleep variability and psychopathology is robust across diverse sociodemographic characteristics.

The finding that general psychopathology is linked to inconsistency in sleep duration has various important theoretical, methodological, and clinical implications. First, current results suggest that fluctuating sleep patterns during adolescence might be a pervasive transdiagnostic mechanism related to nearly all common forms of psychopathology. This notion is supported by our results, which demonstrated that less consistency in sleep duration was related to the general factor of dysregulation and psychopathology (GF-DP) but not the specific internalizing or externalizing factors in the bifactor s-1 model. In addition, supplemental analyses showed that in a correlated factor model, the association between variable sleep duration and psychopathology occurred across internalizing psychopathology, externalizing psychopathology, and emotion dysregulation (ie, with all 3 variables containing common psychopathology/emotion dysregulation variance). Taken together, results suggest that the association between sleep duration variability and psychopathology was truly transdiagnostic, or nonspecific.

Thus, adolescent sleep may be a key target for more parsimonious, efficient, and potentially transformative interventions that could address a broad spectrum of psychiatric disorders with a single treatment. For example, many existing evidence-based treatments for children and adolescents use a modular approach with different therapy modules (eg, exposure, relaxation, rewards) corresponding to different forms of psychopathology (eg, depression, anxiety, conduct problems).^47^ Implementing a dedicated sleep module into already existing modular treatments could not only simultaneously improve various co-occurring psychiatric problems but also enhance the overall efficiency and effectiveness of treatment, by addressing a fundamental and pervasive transdiagnostic factor contributing to psychopathology.

Furthermore, our findings illuminate the potential utility of emphasizing the consistency of sleep as a therapeutic target. Indeed, the Transdiagnostic Intervention for Sleep and Circadian Dysfunction (TranS-C) not only demonstrates significant improvements in sleep outcomes by promoting regular sleep patterns, but also has been shown to ameliorate a wide array of psychiatric symptoms, including internalizing, externalizing, and thought disorder symptoms.^48^ This suggests that interventions targeting sleep consistency align closely with our results and indicate that regularizing sleep schedules could play a pivotal role in a comprehensive strategy to reduce adolescent psychopathology.

In addition, the absence of significant associations between psychopathology and other sleep metrics (ie, average sleep duration, average bedtime and bedtime variability, and average waketime and waketime variability) points to the importance of variability in night-to-night sleep duration as a correlate of transdiagnostic psychopathology. This suggests that focusing solely on averages when assessing sleep quality in this population may not provide a comprehensive picture. Furthermore, it is notable that sleep duration variability, but not variability in waketime or bedtime, was associated with general psychopathology. This suggests that the consistency in how much sleep one is getting is more important for mental health than the consistency of one’s bedtime or waketime. It is possible that the lack of significant associations of waketime and bedtime variability with general psychopathology is related to the lower within-person variability of waketime and bedtime relative to sleep duration, or perhaps is specific to the developmental period of adolescence. Future work is needed with larger sample sizes and across other developmental stages to determine whether sleep duration variability is consistently more important for mental health than variability in bedtime and waketime.

Moreover, the use of MSEM with the MCMC procedure allowed us to capture a more precise and reliable estimate of intraindividual variability than traditional methods (eg, computing the standard deviation across all nights of data for each participant).^46^ Future work interested in relating night-to-night variability in sleep to between-person constructs of interest (eg, psychopathology, personality, physical health) would benefit from using a similar approach. However, given the novelty of the analytic approach, less is known about the minimum number of nights required to provide reliable estimates of sleep variability using MSEM with the Bayesian estimator. We encourage future studies to explore this area further, aiming to refine data collection and analysis standards in sleep research.

Finally, associations between intraindividual variability in sleep duration and the *p* factor did not vary as a function of demographic characteristics. This underscores the robustness of the relationship between sleep variability and psychopathology, suggesting that these findings may have broad relevance across adolescents of different ages, socioeconomic statuses, and sexes. Although the present analysis was powered to detect small effect sizes, the possibility remains that our study may have been underpowered to uncover very subtle moderating effects. Thus, future research with larger sample sizes is encouraged to further explore potential moderation, to ensure the comprehensive understanding and applicability of these findings.

Despite the strengths of this study, including its rigorous methodological approach, several limitations must be noted. First, the study sample’s ethnic and racial composition was predominantly non-Hispanic White, limiting the generalizability of findings to more diverse populations. Second, our cross-sectional design limits our ability to infer the directionality of the observed associations between sleep variability and psychopathology. It is important to acknowledge, as supported by a substantial body of literature, that the relationship between psychopathology and sleep is inherently bidirectional and mutually reinforcing.^20^ Evidence suggests that interventions targeting psychopathology can have beneficial effects on sleep outcomes, just as interventions focusing on sleep improvements can have a positive impact on psychopathological symptoms.^49^^,^^50^ This bidirectionality points to the complex interplay between sleep and psychopathology, highlighting the potential for interventions that concurrently address both domains to offer synergistic benefits. To further elucidate this dynamic relationship, future studies using longitudinal designs and daily assessments of psychopathology and sleep are essential. Such research could provide valuable insights into the day-to-day interactions between sleep patterns and psychopathological states, ultimately informing more effective, comprehensive intervention strategies.

Although the use of a bifactor s-1 model in the present study overcomes many of the limitations of the symmetrical bifactor model, our approach does introduce potential challenges in comparing our findings with those derived from conventional *p* factor analyses because of the unique emphasis on emotion dysregulation. This emphasis enhances the interpretability of the general factor—explicitly characterizing the general factor as emotion dysregulation—but also raises questions about the comparability of our general factor to those identified in other *p* factor studies.^43^ Despite these considerations, previous research with the current sample has shown that the general factor from the bifactor s-1 model, with emotion dysregulation as the reference indicator, shared a nearly identical nomological network with the general factor from a traditionally modeled symmetrical bifactor model that does not include emotion dysregulation as an indicator (Phillips, unpublished data, 2024). This suggests that the general factors derived from these 2 distinct modeling approaches represent largely the same construct, indicating that the choice of modeling strategy may have minimal impact on the general factor’s relationships with other constructs. Furthermore, our factor loadings demonstrate that the general factor was robustly represented across all included forms of psychopathology, underscoring the generality of the general factor.

Consequently, our modeling approach represents a significant advancement by compelling researchers to thoughtfully consider the substantive meaning of their general factor. It underscores the importance of selecting a modeling strategy that not only addresses technical limitations but also enhances theoretical clarity. Future work should prioritize methods, like the one presented here, that allow for a nuanced understanding of the general factor, ensuring that it captures the essence of the construct that it is intended to represent.

In interpreting the present findings, it is essential to consider the observed effect sizes. Consistent with previous research linking sleep variability to psychopathology, effect sizes for associations between intraindividual variability in sleep duration and adolescent psychopathology were small.^27^ However, given the use of 2 fundamentally different methodologies (ie, actigraphy-derived sleep measures, and symptom scales), even a small effect size underscores the robustness of our findings. It is also worth noting that in psychological and clinical research, small effect sizes can still have profound real-world implications, especially when considering the cumulative impact across large populations or over extended periods.^51^ The presence of these effects, despite their small size, highlights the importance of the association between sleep and psychopathology, suggesting that even minor fluctuations in sleep patterns could have ramifications for mental health.

The present study established intraindividual variability in sleep duration as a transdiagnostic factor, related to the general factor of dysregulation and psychopathology, during the critical developmental period of adolescence. The absence of significant demographic moderators hints at the potential broad relevance of understanding sleep variability as a key factor in adolescent psychopathology. Our findings not only challenge traditional, disorder-specific approaches but also align with and contribute to a burgeoning evidence base advocating for a transdiagnostic perspective in both research and clinical practice.^3^^,^^52^ This calls for a paradigm shift in how we conceptualize and treat adolescent psychopathology, underscoring the need for more comprehensive approaches that can address a broad range of psychiatric conditions. By targeting sleep variability, clinicians and researchers may be able to design more effective, comprehensive interventions that could have a profound impact on the mental health trajectory of adolescents, potentially reducing the prevalence of a broad spectrum of psychiatric disorders across the lifespan.

## CRediT authorship contribution statement

**Eric M. Phillips:** Writing – review & editing, Writing – original draft, Methodology, Formal analysis, Conceptualization. **Emily L. Goldberg:** Writing – review & editing, Writing – original draft, Data curation, Conceptualization. **Rebecca L. Brock:** Writing – review & editing, Methodology, Formal analysis, Conceptualization. **Emily R. Hamburger:** Writing – review & editing, Methodology, Data curation. **Jennifer Mize Nelson:** Writing – review & editing, Methodology. **W. Alex Mason:** Writing – review & editing, Funding acquisition, Conceptualization. **Kimberly Andrews Espy:** Writing – review & editing, Funding acquisition. **Timothy D. Nelson:** Writing – review & editing, Writing – original draft, Supervision, Methodology, Conceptualization.

## Supplemental Material

Supplemetal Table
