# Supplementary material for: Adolescent Sleep as a Transdiagnostic Factor: Associations Between Actigraphy-Derived Night-to-Night Sleep Metrics and Adolescent Psychopathology
Source: JAACAP Open. 2024 Jul 4;3(3):701–12. doi: 10.1016/j.jaacop.2024.06.001 (PMC12414309; doi:10.1016/j.jaacop.2024.06.001)
Supplement: Supplemetal Table [file mmc1.docx]

| *Table S1: Demographic Characteristics for Participants with Valid versus Missing Sleep Data* | | | | | |
| --- | --- | --- | --- | --- | --- |
| **Demographic Characteristics** | **Valid Sleep Data** | | **Missing Sleep Data** | | **Difference** |
|  | **Mean** | **SD** | **Mean** | **SD** | **p-value** |
| Age | 15.33 | 1.139 | 14.85 | 1.048 | 0.022 |
| Maternal Education | 15.47 | 2.320 | 15.32 | 2.345 | 0.732 |
| Income to Needs Ratio | 2.46 | 1.733 | 2.96 | 2.374 | 0.150 |
|  | **n** | **(%)** | **n** | **%** |  |
| **Sex (Assigned at Birth)** |  |  |  |  | 0.057 |
| Male | 90 | 44.1 | 21 | 61.8 |  |
| Female | 114 | 55.9 | 13 | 38.2 |  |
| **Ethnicity** |  |  |  | | 0.453 |
| Not Hispanic or Latino | 177 | 86.8 | 31 | 91.2 |  |
| Hispanic or Latino | 27 | 13.2 | 3 | 8.8 |  |
| **Race** |  | |  | |  |
| Asian | 1 | 0.5 | 0 | 0.0 | 0.681 |
| Black or African American | 8 | 3.9 | 2 | 5.9 | 0.618 |
| Multiracial | 49 | 24.0 | 4 | 11.8 | 0.127 |
| White | 146 | 71.6 | 28 | 82.4 | 0.208 |
|  |  | |  | |  |
| **Key Study Variables** |  | |  | |  |
|  | **Mean** | **SD** | **Mean** | **SD** |  |
| General Factor | 0.021 | 0.954 | -0.077 | 0.936 | 0.579 |
| Specific Internalizing Factor | -0.027 | 0.846 | 0.066 | 0.955 | 0.560 |
| Specific Externalizing Factor | 0.020 | 0.867 | -0.066 | 0.731 | 0.594 |
